# The Role of Observation–Measurement Methods in the Surface Characterization of X39Cr13 Stainless-Steel Cutting Blades Used in the Fish Processing Industry

**DOI:** 10.3390/ma13245796

**Published:** 2020-12-18

**Authors:** Wojciech Kapłonek, Krzysztof Nadolny, Bartosz Zieliński, Jarosław Plichta, Danil Yurievich Pimenov, Shubham Sharma

**Affiliations:** 1 Department of Production Engineering, Faculty of Mechanical Engineering, Koszalin University of Technology, Racławicka 15-17, 75-620 Koszalin, Poland; wojciech.kaplonek@tu.koszalin.pl (W.K.); jaroslaw.plichta@tu.koszalin.pl (J.P.); 2 Espersen Koszalin Sp. o.o., Mieszka I 29, 75-124 Koszalin, Poland; b.zielinski@espersen.com; 3 Department of Automated Mechanical Engineering, South Ural State University, Lenin Prosp. 76, 454080 Chelyabinsk, Russia; danil_u@rambler.ru; 4 Department of Mechanical Engineering, IK Gujral Punjab Technical University, Jalandhar-Kapurthala Road, Kapurthala 144603, Punjab, India; shubham543sharma@gmail.com

**Keywords:** observation–measurement methods, image processing and analysis, surface characterization, cutting blade, regeneration, precision grinding

## Abstract

In the modern fish processing industry, flat fishes play an important role. They are processed into a final product in the form of a fillet during the skinning operation, which is carried out on machines operating in automated production lines. These machines are usually equipped with a single planar cutting blade or a few of such blades. The high-efficiency skinning and industrial conditions cause rapid wear of the cutting edge of the blade, which is detrimental to the quality of the final product. One of the forms of renewing the cutting ability of these types of tools is the regeneration carried out with the use of precise traverse surface grinding. The results of this process must be carefully verified for determining its correctness and possible optimization of its parameters. The main goal of this article was to characterize the usefulness of a number of observational and measuring methods to evaluate the results of the technical blade regeneration process. In this work, a number of contemporary observation–measurement methods such as optical microscopy (OM), scanning electron microscopy (SEM), energy-dispersive X-ray spectroscopy (EDS), optical profilometry (OP), and angle-resolved scattering (ARS), supported by image processing and analysis techniques, were analyzed. The authors focused on presenting the role of the abovementioned methods in the surface characterization of planar cutting blades made of X39Cr13 chromium martensitic stainless steel before and after the technological operation of flat-fish skinning. Additionally, the surface condition after the regeneration process carried out using the five-axis CNC (computerized numerical control) grinding machine was also assessed. Numerous results of surface observations, elemental composition microanalysis, high-accuracy surface microgeometry measurements, and quantitative and qualitative analysis confirming the possibility of using the proposed methods in the presented applications are presented.

## 1. Introduction

Fish has been an important component of the human diet since ancient times, containing 63–78% water, 15–20% protein, 1–30% fat (lipids), and about 0.1% saccharides. Among several thousand fish species, only about 350 are of industrial importance. In Poland, fish products from marine and freshwater fish are mainly consumed. The former are obtained mainly from Polish sea areas, the total area of which is 32,400 km^2^ (including the area of the territorial sea—8681 km^2^), as given by Kapusta [1]. The fisheries in the Baltic Sea are considered to be relatively rich in fish resources, the catches of which are mainly focused on the fishes from the Clupeidae family (Baltic herring (*Clupea harengus membras*) and Baltic sprat (*Sprattus sprattus balticus*)), Gadidae family (Baltic cod (*Gadus morhua callarias*)), and Pleuronectidae family (Baltic plaice (*Platessa platessa baltica*)). The latter, classified as a flat fish, widely presented by Gibson et al. [2], is the fourth most fished species in this area. As reported by Kapusta [1], fishing levels in 2011 and 2012 were 9725 t and 10,089 t, respectively (for the European flounder (*Platichthys flesus trachurus*). In the same period, the level in Great Britain was 4000 t (for the European plaice (*Pleuronectes platessa*)), as reported by Lart and Green [3]. The basic systematic information and general morphological characteristics of European plaice (*Pleuronectes platessa*) are given in Table A1 and Table A2 (Appendix A), respectively.

The fillet obtained during skinning is particularly desirable. It is an operation, comprehensively characterized by Hall [4,5], that allows removal of undesirable elements from the fish raw material [6], as well as providing it with appropriate dimensions and shape. This operation focuses on the effective separation of the soft tissues and can be carried out in two variants—basic/intensive skinning (consisting of the removal of the skin without/with the silver tissue on the fillet).

**Figure 1:**
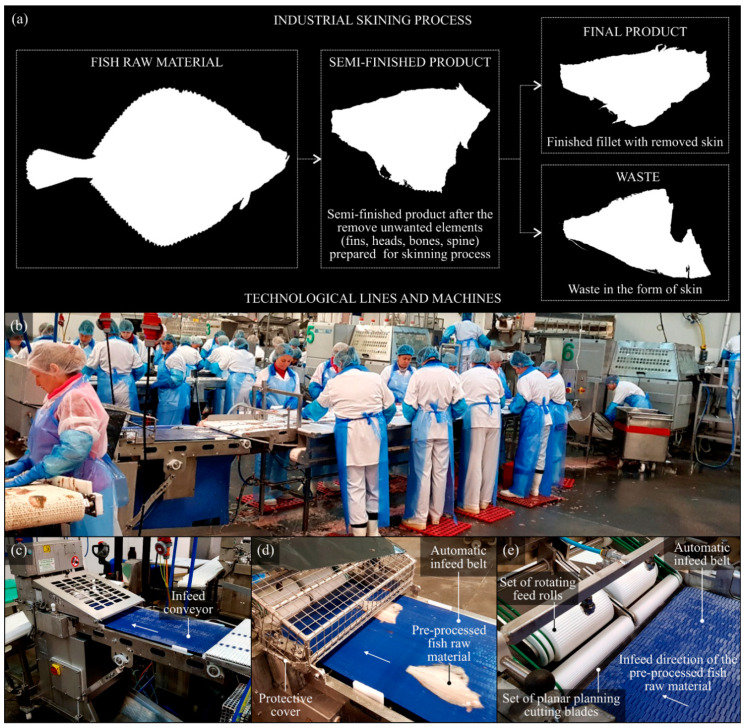
The production process carried out on the technological line for the processing (skinning) of flat fishes from *Pleuronectidae* family, based on the example of the a medium-sized fish processing plant (Espersen Koszalin Sp. z o.o., Koszalin, Poland): (**a**) general diagram of the industrial skinning process; (**b**) general view of one of the technological lines; (**c**)automatic skinning machine ST600 (Steen F.P.M. International, Kalmthout, Belgium); (**d**) close-up of the infeed conveyor a pre-processed raw fish material; (**e**) close-up of the set of planar cutting blades mounted under the rotating feed rolls.

In industrial conditions, flat-fish processing is carried out using a wide range of technological machines, most often operating in an automatic mode, as presented by Caldwell [7], Bar [8], Tomczak-Wandzel et al. [9], and Kamaruzzaman et al. [10]. Fish-skinning machines (stationary or benchtop) use various types of planar cutting blades. Depending on the construction of the machine, its configuration, and its use in a given technological operation, it can be equipped with a single blade or a few blades (in the form of a multiblade head), which, to a large extent, allows for increasing the efficiency of processing raw fish material. The tool or tools used for separating the raw material can realize a stationary or reciprocating motion in relation to the feedstock. Figure 1 illustrates the above issues concerning the processing of flat fish under industrial conditions, using the example of a medium-sized fish processing plant in Koszalin (Poland). A general diagram of the industrial skinning process (from the introduction of the fish raw material to the process to the final product) is shown in Figure 1a. A fragment of one of the technological lines for the skinning operation of flat fish from the Pleuronectidae family, mainly the flounder (*Platichthys flesus trachurus*) and plaice (*Platessa platessa baltica*), is presented in Figure 1b. One of the many types of skinning machines used, in this case, ST600 (Steen F.P.M. International, Kalmthout, Belgium), is shown in Figure 1c. The general view allows acquainting oneself with the construction of this technological machine and its components, which are presented in detail in the close-ups shown in Figure 1d,e. The ST600 is a stationary machine characterized by high efficiency and quality of the skinning involving a high-speed, dual-lane infeed of raw fish material and minimal pressure on the fillet.

As stated above, the planar cutting blades are the essential tool used to process raw fish material. Depending on the specificity of the processed fish species, the type of technological machines, and the process parameters, blades with specific properties are used. They are produced by many types of carbon and alloy tool steels, as well as high-grade stainless steel. Of particular importance in this respect are cryogenically hardened stainless steels with a polished surface (e.g., X39Cr13, X65Cr13, and X65CrMo14). In many cases, blade manufacturers further improve the cutting edges by using various coatings, well characterized by Bobzin [11]. The coatings are deposited via physical vapor deposition (PVD) [12,13,14] or chemical vapor deposition (CVD) [15,16,17,18], while the typical coatings used on these types of cutting tools are TiN, TiC, TiCN, TiAlN, ZrN, DLC, CrN, and Teflon^®^.

The mentioned coatings provide a relative extension of the tool life. However, the progressive wear of the cutting edge of the planar planning cutting blades after some time will lead to a situation in which the technological process cannot be effectively conducted. The source here is unfavorable factors occurring in production conditions related to adverse corrosive interactions (carrying out a process in high-humidity conditions, use of chlorine-based washing preparations, and strong nitric (NHO_3_)/phosphoric (H_3_PO_4_)-based acids) and/or mechanical (abrasive) wear due to changes in tool geometrical dimensions (edge dulling, cavities, cracks, scratches, etc.) caused by geometry variation of the fish raw material, it being incorrectly set on the feeder, inadequate removal of local impurities (small-sized sand grains, slight shells and fish scales, etc.), and other impurities transferred from previous processes.

The significant impact of these factors causes degradation of the cutting tool and reduces its ability to separate soft tissues. This entails a reduction in the efficiency of the technological process and the final product’s quality, as discussed by Sampels [19]. A critical moment is a replacement of blades (e.g., in a multiblade head), leading to a temporary or a long-term stopping of technological machines operating in the production line. Downtime generates costs and can cause financial losses. The problem of extending the tool life, presented among others by Bermingham et al. [20], Yamnikov et al. [21], and Maegawa et al. [22], is one of the fundamental issues related to industrial production. In relation to the planar cutting blades, one of the proposed solutions is the effective regeneration of the tool (obtaining its original shape and recreating the correct inclination angles of cutting edges). An original proposal of this type of regeneration, using a precise grinding process by grinding wheels (regular boron nitride (cBN) [23,24,25] abrasive grains bonded by ceramic binder) attached to an electro-spindle of the five-axis CNC (computerized numerical control) grinding machine, was presented by Zieliński et al. [26,27]. In case of realization of this process, special attention should be paid to thermal conditions in the grinding wheel with the machined surface contact zone. A thin planar cutting blade heats up easily, which can result in undesired structural changes (grinding defects). For this reason, it is necessary to use cBN grinding wheels characterized by a high heat conduction coefficient. In addition, it is recommended to use higher grinding speeds because, under such conditions, the vast majority of the heat generated in the grinding zone is transferred to the chip and removed from the machining zone with it.

The presentation of selected issues related to the above problem in a slightly broader approach is proposed in this article with emphasis on the role of observation and measurement methods in characterizing cutting tools regenerated by precision abrasive machining methods. In Section 2, the selection and characteristics of the assessed planar cutting blades (Section 2.1), the general characteristics of machining (regeneration) conditions (Section 2.2), and detailed information on the type and configuration of the observation–measurement instruments used are given (Section 2.3). In Section 3, an example of the results of the experimental studies is described and carefully discussed. The results are presented related to microscopy observations of the raw fish material impurities along with quantitative analysis of the SiO_2_ grains (Section 3.1), microanalysis of the elemental composition of the planar cutting blade’s material (Section 3.2), high-accuracy surface microgeometry measurements of the planar cutting blade after the technological process (skinning operation) (Section 3.3), electron imaging of cutting edge surface and cutting products before and after the regeneration process (Section 3.4), and imaging and analysis of scattered light from the regenerated surface of the planar cutting blade (Section 3.5). In Section 4, the significant conclusions and perspectives for further work in this area are presented.

## 2. The Methodology of Experimental Studies

### 2.1. The Planar Cutting Blades Selected for Experimental Studies

For the experimental studies, a set of seven planar cutting blades (Kuno Wasser GmbH, Solingen, Germany for Steen F.P.M. International, Kalmthout, Belgium) made for X39Cr13 high-carbon martensitic stainless steel were selected. The cutting tools were mounted in automatic skinning machine Steen ST600 and used during the skinning operation of the flat fishes (flounder (*Platichthys flesus trachurus)* and European plaice (*Pleuronectes platessa*) obtained from Baltic fisheries). After an incidental finding of critical wear of the cutting edge by an employee, the blades are intended for the regeneration process. Basic information on the planar cutting blades is given below.

**Figure 2:**
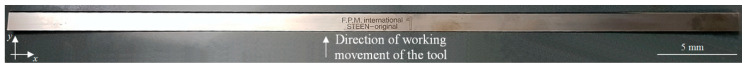
Example planar cutting blade used in the experimental studies.

In Figure 2, the general view of an example cutting tool is presented. The dimensional characteristics of the planar cutting blade with elemental composition of its material (X39Cr13) are given in Table 1. The physical and processing properties of X39Cr13 are given in Table 2.

### 2.2. Characteristics of the Regeneration Process of the Planar Cutting Blades

**Figure 3:**
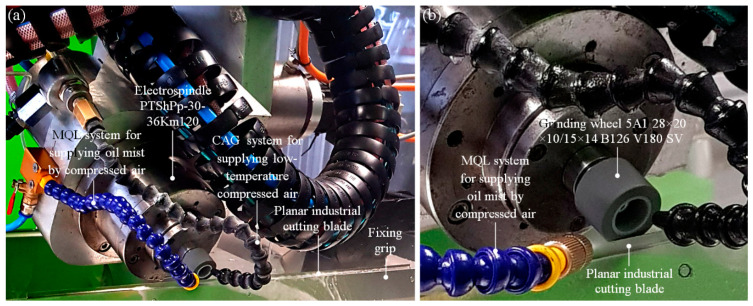
Central part of a five-axis CNC (Computerized Numerical Control) grinding machine: (**a**) general view; (**b**) close-up of the grinding wheel 5A128 × 20 × 10/15 × 14B126V180SV mounted in electro-spindle PTShPp-30-36Km120

The regeneration process of cutting edge of all selected planar cutting blades was realized using a five-axis CNC grinding machine, described in detail by Zielinski [26]. As shown in Figure 3a, the central part of the technological machine contains an electro-spindle PTShPp-30-36Km120 (FŁT-Kraśnik S.A., Kraśnik, Poland) with a grinding wheel 5A128 × 20 × 10/15 × 14B126V180SV (Inter-Diament, Grodzisk Mazowiecki, Poland); the machining conditions are given in Table 3.

### 2.3. Characteristics of the Observation–Measurement Instruments Used during Experimental Studies

During experimental studies, the authors used many types of observation–measurement instruments. Their detailed information, covering the system type, designation, producer, and components and features of the given instrument, is presented in Table 4.

## 3. Results and Discussion

### 3.1. Observations of the Raw Fish Material Impurities and Quantitative Analysis of the SiO_2_ Grains

**Figure 4:**
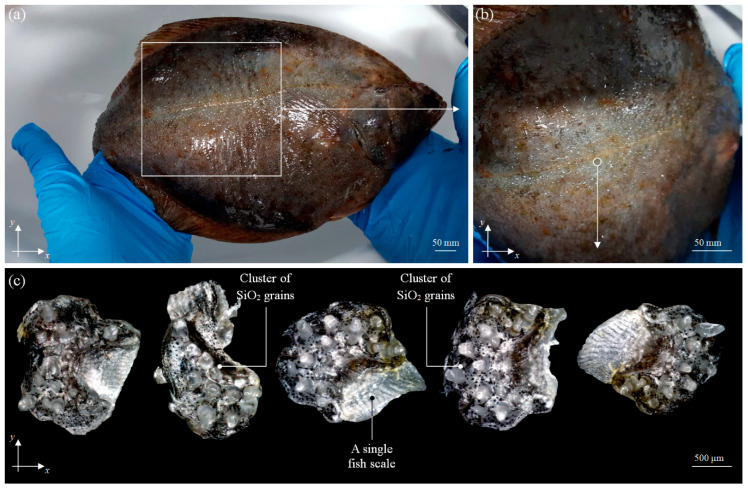
European plaice ♂ (*Pleuronectes platessa*): (**a**) general view of the specimen before the processing; (**b**) close-up of part of the body with a small-sized randomly located SiO_2_ grain clusters; (**c**) examples of extracted fish scales with visible SiO_2_ grain clusters.

The raw fish material must be properly prepared for the technological process. Leaving aside the general phase of washing, one of the most important steps is removing all types of impurities that may affect the process (especially during mechanical (abrasive) processing). Great importance is attached to removing scales and grains of sand, which may be one of the causes of the rapid wear of cutting edges of the planar cutting blades operating in skinning machines. Grains of sand (SiO_2_) often appear on flat fish because of its demersal life, on both sides of the body (during the hunt, the dorsal part of the body is also covered with a small sand layer, which acts as camouflage). Figure 4a shows a European plaice ♂ (*Pleuronectes platessa*), 460.73 mm long, obtained from Baltic fisheries.

The close-up of its dorsal part of the body revealed numerous randomly arranged small-sized randomly located SiO_2_ grain clusters located on individual scales, as shown in Figure 4b. Fifty scales with visible groups of SiO_2_ grains were taken from the specimen; using an optical stereo zoom microscope SSM-EC2 (Schut Geometrische Meettechniek B.V., Groningen, The Netherlands) equipped with a color CMOS (Complementary Metal–Oxide–Semiconductor) camera MicroCam 10 MP (Bresser GmbH, Rhede, Germany), images were acquired. Examples of the results are shown in Figure 4c.

Using Image-Pro^®^ Plus 5.1 (Media Cybernetics, Inc., Rockville, MD, USA) [29,30,31] software, quantitative analysis of the SiO_2_ grain clusters was carried out. During analysis, the average values of selected geometrical parameters (*An*, *p*, *l*, *w*, *F_min_*, *F_max_*) were calculated for *n* = 50 samples. The results were as follows: area *An* = 6.06 mm^2^, perimeter *p* = 0.65 mm, size (length) *l* = 0.23 mm, size (width) *w* = 0.16 mm, and Feret (max. and min. values) *F_min_* = 0.22 mm, *F_max_* = 0.21 mm. In the observed clusters, there were 912 grains outside the clusters, and there were usually single grains. Despite the relatively low height (between 50 and 500 μm), the grains were an important source of impurities occurring in the raw fish material, directly affecting the condition of the cutting edge of the tool.

### 3.2. EDS Quantitative Microanalysis of the Elemental Composition of the Planar Cutting Blade’s Material

**Figure 5:**
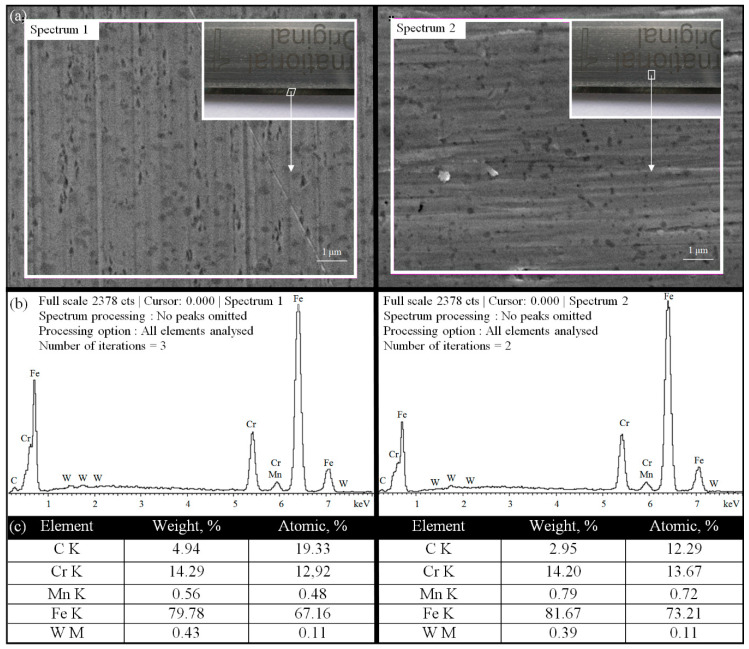
Energy-dispersive X-ray spectroscopy (EDS) quantitative microanalysis of the surface of the planar cutting blade (reference): (**a**) SEM micrograph of two selected areas (11.07 × 8.27 μm) for which a microanalysis was carried out; (**b**) conditions of EDS microanalysis and EDS spectra obtained with identified elements contained in the analyzed areas; (**c**) calculated weight and atomic percentages of elements.

To precisely identify the chemical elements included in the material from which planar cutting blades were made and to compare them with the composition of X39Cr13 high-carbon martensitic stainless steel, an energy-dispersive X-ray spectroscopy (EDS) quantitative microanalysis was carried out. For this purpose, the dispersive spectrometer module INCAPenta FET-x3 (Oxford Instruments, Abingdon, Great Britain) coupled with JSM-5500 LV (JEOL Ltd., Tokyo, Japan) [32,33] was used. The EDS quantitative microanalysis was carried out for a random area located at the cutting edge (Spectrum 1) and body (Spectrum 2) of the reference planar cutting blade. The results obtained during EDS quantitative microanalysis are shown in Figure 5.

The obtained results confirmed the high content of elements such as Fe, Cr, and C, with a trace of Mn, in the analyzed sample. The obtained weight percentages, analyzed for two key elements, iron and chromium (Fe_S1_ = 79.78%, Fe_S2_ = 81.67% and Cr_S1_ = 14.29%, Cr_S2_ = 14.20%, respectively), were consistent with the values given in Table 1 (Fe = 83.00–87.00% and Cr = 12.50–14.50%, respectively). The above values also confirmed the high consistency of the obtained results and, in the case of the key elements Fe, Cr, and Mn, clearly indicated X39Cr13 high-carbon martensitic stainless steel as the material from which the planar cutting blades were made.

### 3.3. Surface Microgeometry Measurements of the Planar Cutting Blades after the Technological Process

As stated earlier, the intensive skinning operation (as part of a high-efficiency technological process) causes relatively rapid wear of the cutting edge of the planar cutting blade. Additionally, the appearance of impurities from previous processes (including SiO_2_ grains—Section 3.1) may intensify this effect. The analysis of the cutting edge surface microgeometry can be directed, among others, to assess the condition of its wear. In the carried out experimental studies, such an analysis was realized on all planar cutting blades obtained from the automatic skinning machine Steen ST600 after the technological process. Measurement data for analysis were obtained using a multisensory optical profilometer Talysurf CLI2000 (Taylor-Hobson, Leicester, Great Britain) equipped with a laser triangulation sensor LK-031 (Keyence Corp., Osaka, Japan) [34,35].

**Figure 6:**
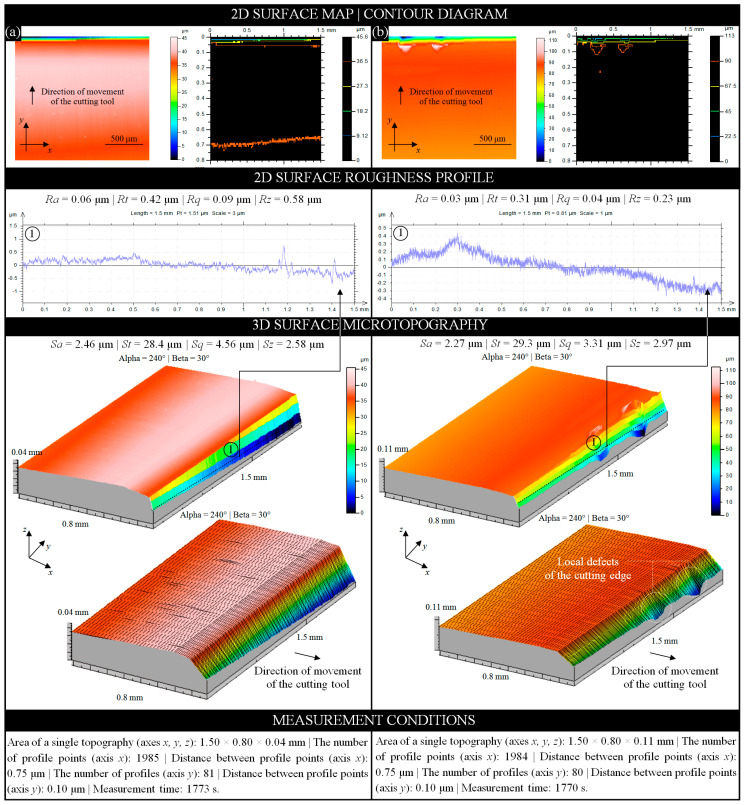
Collection of selected results of experimental studies obtained using multisensory optical profilometer Talysurf CLI2000 (Taylor-Hobson, Leicester, Great Britain) for a fragment of the cutting edge surface (1.5 × 0.8 mm) of planar cutting blade: (**a**) for sample B1-4; (**b**) for sample B1-5.

Figure 6 presents example analyses prepared in TalyMap Silver 4.1 software using Mountains Technology^®^ (Digital Surf, Besançon, France) for two planar cutting blades. The samples were selected to show different forms of wear. Figure 6a presents the analyses obtained for sample B1-4 in the form of a two-dimensional (2D) surface map (indexed colors), contour diagram (color curves mode, indexed colors), and 3D surface microtopography (continuous and meshed axonometric mode) with a single extracted 2D surface roughness profile. The above analyses were supplemented with the determined values of selected basic roughness (profile; *Ra*, *Rt*, *Rq*, and *Rz*) and amplitude (surface; *Sa*, *St*, *Sq*, and *Sz*) parameters. The conditions under which the measurements were performed are given at the bottom of the Figure 6a.

Sample B1-4, despite the visual absence of defects, had strong frontal wear of the cutting edge caused by the intense separation of soft tissues. The values of roughness (profile) and amplitude (surface) parameters showed a significant reduction (on average 40% and 24%, respectively) compared to the values obtained for the reference sample (new and unused planar cutting blade). For the roughness (profile) parameters, the lowest value (19.44%) was obtained for the *Rz* parameter (*Rz* = 0.58 μm), while the highest value (55.78%) was obtained for the *Rt* parameter (*Rt* = 0.42 μm). For the amplitude (surface) parameters, the lowest value (25.43%) was obtained for the *Sz* parameter (*Sz* = 2.58 μm), while the highest value (37.08%) was obtained for the *Sa* parameter (*Sa* = 2.46 μm).

The same analyses (as for sample B1-4) were prepared for sample B1-5 and are presented in Figure 6b. Sample B1-5, despite a much smaller frontal wear of the cutting edge, was characterized by the presence of two defects in this area. Their shape indicated a loss of material due to the action of a small-sized SiO_2_ grains found on the processed raw fish material during the skinning operation. As in the previous case, the values of roughness (profile) and amplitude (surface) parameters showed a significant reduction (on average 70% and 32%, respectively) compared to the values obtained for the reference sample. For the roughness (profile) parameters, the lowest value (67.36%) was obtained for the *Rt* parameter (*Rt* = 0.31 μm), while the highest value (73.33%) was obtained for the *Rq* parameter (*Rq* = 0.04 μm). For the amplitude (surface) parameters the lowest value (14.16%) was obtained for the *Sz* parameter (*Sz* = 2.97 μm), while the highest value (46.95%) was obtained for the *Sq* parameter (*Sq* = 2.93 μm).

### 3.4. SEM Imaging of Cutting Edge Surface and Cutting Products before and after the Regeneration Process

The condition of example cutting edges of the planar cutting blades after the technological process, considered in the previous subsection from a surface geometry point of view, could also be visually represented by imaging realized using scanning electron microscopy (SEM). This technique allows for the precise observation of selected fragments of the surface in a wide range of magnifications to analyze the forms and intensity of its wear. The scanning electron microscope JSM-5500 LV (JEOL Ltd., Tokyo, Japan) was used for imaging (with magnification from 200× to 900×) of interesting fragments of the cutting edges worn surface.

**Figure 7:**
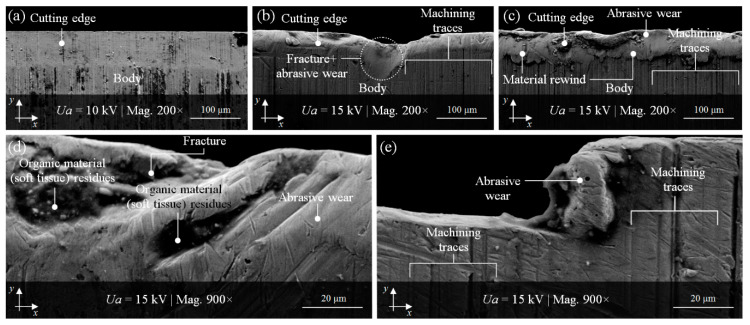
Selected results of SEM imaging (JEOL JSM-5500LV) of planar cutting blades after the intensive technological process: (**a**) nonworn cutting edge (reference, magnification (mag.) 200×); cutting edges with visible various degrees of degradation: (**b**) B1-2 (mag. 200×); (**c**) B1-3 (mag. 200×); (**d**) B1-3 (mag. 900×); (**e**) B1-7 (mag. 900×).

Figure 7b–e show examples of various degrees of degradation of selected cutting edges (B1-2, B1-3, B1-7) after the intensive technological process with reference to a nonworn cutting edge (reference) presented in Figure 7a. In this figure, the separation between the cutting edge area and the body area of the reference planar cutting blade is clearly visible with numerous machining traces.

Perpendicular to the cutting edge, machining traces are also clearly visible in Figure 7b,c,e, as well as partially in Figure 7d. The abrasively worn cutting edge of sample B1-2 presented in Figure 7b had a 87.61 μm diameter fracture in the central part, while, in Figure 7c, the material rewinding is perfectly visible. The higher magnification (900×) revealed more details. Figure 7d shows a fragment of the sample B1-3 with cavities in which the technological process residues (fragments of separated organic material—soft tissue) are visible. Figure 7e presents a magnified image (900×) of a fragment of sample B1-7with strong abrasive wear of the cutting edge.

All of the planar cutting blades, whose condition after the technological process was presented in the previous subsection, were subjected to regeneration. This process, described in detail by Zieliński et al. [26,27], consisted of renewing the cutting ability—restoring the original shape and recreating correct cutting edges angles of inclinations. The effects of the regeneration were verified using high-accuracy contact (stylus) [27] and noncontact (optical) [26] measurement systems for determining its correctness. Numerous parametric analyses confirmed the possibility of this type of regeneration carried out using the precision traverse surface grinding process (in peripheral and front grinding kinematics). However, it was not optimal for all of the machined planar cutting blades’ edges. Much less favorable results were obtained for peripheral grinding process realized at *v_w_* = 300–400 mm/min and *n_s_* = 38,000 min^−1^.

**Figure 8:**
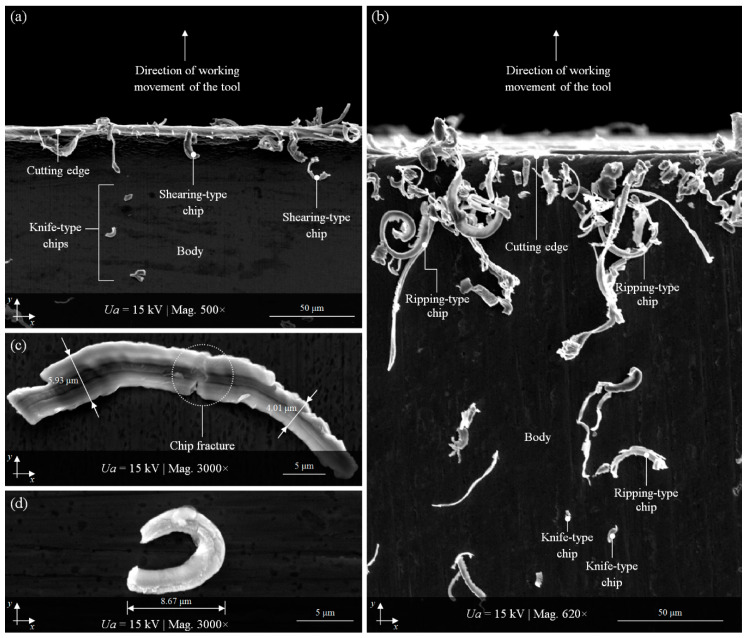
Selected results of SEM imaging (JEOL JSM-5500LV) of planar cutting blade B1-3 immediately after regeneration process: (**a**,**b**) a vast panorama of a fragment of the sample surface with a visible cutting edge, body, and numerous flow-type chips (mag. 500× and 620×, respectively); (**c**) close-up of a shearing-type chip (mag. 3000×); (**d**) close-up of a knife-type chip (mag. 3000×).

For one of the samples (B1-3) ground with these kinematics, imaging (with magnification from 500× to 3000×) of the cutting-edge surface and cutting products (flow-type chips) was obtained by scanning electron microscope JSM-5500LV (JEOL Ltd., Tokyo, Japan). Some examples of the results obtained for this sample are shown in Figure 8. Figure 8a,b offer a vast panorama of a fragment of the B1-6 sample surface with a visible cutting edge and body immediately after the regeneration process. The cutting edge was not yet devoid of machining products (they were left on purpose for later observation); after regeneration, it was always thoroughly cleaned and washed. In this case, different types of flow-type chips can be observed. These include ripping-type, shearing-type, and knife-type chips. All of these subtypes are visible in Figure 8a,b. The morphology of shearing-type and knife-type chips is depicted in more detail in Figure 8c,d, respectively.

### 3.5. Imaging and Analysis of Scattered Light from the Regenerated Surface of the Planar Cutting Blades

The results of the parametric microgeometry analysis of regenerated planar cutting blade surfaces, obtained using high-accuracy measurements with a stylus and optical profilometry, can be extended to other observation–measurement techniques. Due to the laboratory characteristic of these methods, their application in industrial conditions is highly limited. Therefore, a new promising method, which can be an alternative to those mentioned above, is still desired. The most interesting are those that can be used to carry out the measurement process in specific conditions characterized by the presence of various types of contamination (dust), high air humidity, and generation of mechanical vibrations. The pursuit of measurements at the production process site is related to an essential trend in the modern processing industry. The integration of the technological process with the measurement process brings many benefits and directly affects the final product’s quality. One such interesting method that meets the rigorous industrial conditions is angle-resolved scattering (ARS). This method is based on imaging and analysis of the angular distribution of the scattered light intensity. ARS was used in the past by authors in numerous research works for the quick and noncontact inspection of surface irregularities and localization of their various type of defects. Some of the results of these studies were described by Kapłonek et al. [36], Kapłonek and Nadolny [37], and Kapłonek et al. [31].

The verification of the regeneration process was carried out using an experimental setup for the acquisition of images of scattered light intensity, discussed in detail in the above publications. For the illumination of planar cutting blades’ cutting edges, a laser diode Lasiris™ SNF 635 (Coherent Inc., Santa Clara, CA, USA) was used. The laser beam (wavelength λ = 635 nm) illuminated the assessed surface of each of the samples at an angle of incidence of 50°. The scattered light from the surface was incident to a 300 × 300 mm white matte screen with a scale pattern. The image from the observation plane was acquired using a Camedia C-5060WZ (Olympus, Tokio, Japan). The camera was mounted on an antivibration platform. The control of the acquisition process was realized by remote cable release RM-UC1.

**Figure 9:**
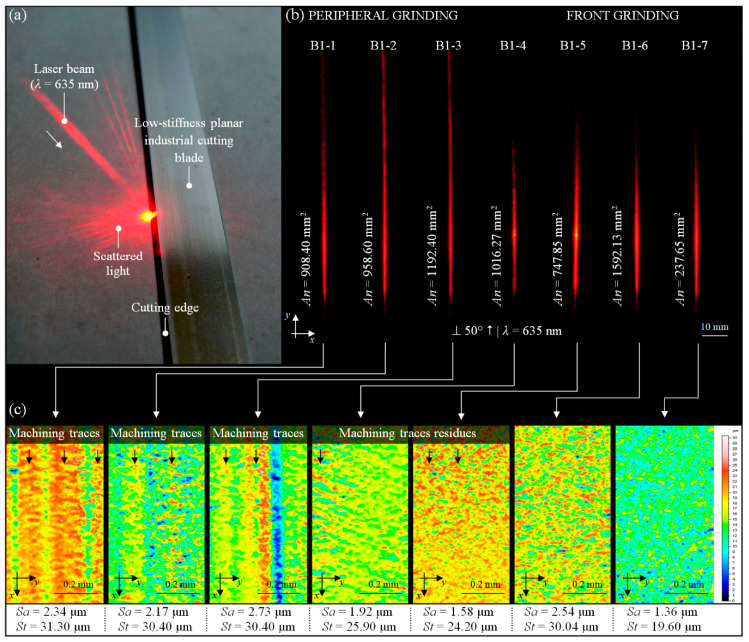
Collection of selected results of experimental studies obtained for cutting edge of the planar cutting blades: (**a**) regenerated sample B1-5 illuminated by a laser beam (λ *=* 635 nm); (**b**) acquired images of the angular distribution of scattered light intensity (B1-1–B1-7) with calculated average values of the area using Image Pro^®^-Plus 5.1 (Media Cybernetics Inc., Rockville, MD, USA) software; (**c**) corresponding with images from Figure 9b, the 2D surface maps obtained on the basis of of measurement data from a multisensory optical profilometer Talysurf CLI2000 (Taylor-Hobson, Leicester, Great Britain) with calculated amplitude (surface) parameters.

The acquired images of the angular distribution of scattered light intensity were processed and analyzed in a specialized image processing and analysis environment—Image Pro^®^-Plus 5.1 (Media Cybernetics Inc., Rockville, MD, USA). The parametric analysis included the determination of the values of parameters characterizing the obtained distributions of scattered light. A more detailed description of the following phases of the analysis was given by Kapłonek [31] and Kapłonek et al. [36]. In Figure 9, example results of experiments carried out are presented.

A fragment of the regenerated cutting edge of the planar cutting blade B1-5 illuminated by the beam (λ = 635 nm) from the laser source is shown in Figure 9a. The images of the angular distribution of scattered light intensity, acquired for all of the analyzed samples (B1-1–B1-7), are presented in Figure 9b. A visual analysis of these distributions shows their differences in the case of peripheral and front grinding. A visual analysis of these distributions showed their differences in the case of peripheral and front grinding. The second kinematic obtained more favorable results, which was also confirmed by the values of *An* (area of the bright regions of an image of scattered light). The average area value (*An_avr_* = 1019.80 mm^2^) calculated for samples regenerated by peripheral grinding was higher than that for samples regenerated by front grinding (*An_avr_* = 898.47 mm^2^). For sample B1-7, the lowest value (*An* = 237.65 mm^2^) was obtained, while, for sample B1-6, the highest value was obtained (*An* = 1592.13 mm^2^). The 2D surface maps, corresponding with images of the angular distribution of scattered light intensity from Figure 9b, are presented in Figure 9c. The 2D surface maps in indexed colors (height of the surface elements encoded with the given color) were generated in TalyMap Silver 4.1.2 (Digital Surf, Besançon, France) software on the basis of measurement data obtained using a multisensory optical profilometer Talysurf CLI2000 (Taylor-Hobson, Leicester, Great Britain). Furthermore, for comparison purposes, the figure also includes the calculated values of the amplitude (surface) parameters—*Sa* and *St*. These values indicate much better results obtained for face grinding, which is a more efficient process than peripheral grinding.

## 4. Conclusions

This article discussed the important role of observation–measurement methods in an interesting application concerning the analysis of cutting edges of planar cutting blades used in the modern fish processing industry. Particular methods proposed by the authors were shown against the background of the experiments that were carried out on the abovementioned cutting tools before and after the technological process (skinning operation), as well as after the regeneration process (precise grinding). The obtained results of the experimental studies gave a basis for formulation of the following conclusions:

- Proper verification of the correctness of the planar cutting blade regeneration process realized using CNC grinding machines requires the use of observation–measurement methods allowing for imaging, measuring, and analyzing the assessed surfaces for determining the correctness of the renewing process.
- Electron imaging, as well as stylus and optical profilometry, can be used in this particular application, and it is recommended that they should be supported by specialized, dedicated software (e.g., TalyMap, Image Pro^®^-Plus) for measurement data processing (often large volumes) and analysis, e.g., correction/filtering, geometric measurements of measured/imagined elements, parametric analyses, and appropriate presentation (visualization) of obtained results.
- The indicated selection of observation–measurement methods resulted mainly from the need to unequivocally assess the traces of both abrasive wear and durability wear caused by contact with foreign bodies anchored in the processed material, resulting from, e.g., the living environment of flat fishes, which remain mainly on the seabed.
- In addition to the universal laboratory observation–measurement methods, an interesting solution is the use of light scattering methods, such as angle-resolved scattering, which was presented in Section 3.5. Due to their industrial characteristics, they can be used both on the production line (e.g., in in-process inspection systems as part of an automatic skinning machine) and during the regeneration process (e.g., in precise in-process inspection systems mounted on the CNC grinding machine).
- The positive results of the experimental studies have motivated the authors to continue further research work on this interesting subject in the near future. Activities are planned, among others, related to the technological direction (further improvement of the design of the CNC grinding machine; further optimization of the parameters of the cutting-edge regeneration process by means of precision grinding) and metrological direction (analysis of possibilities of using other observational and measuring methods than those presented in this article; analysis of the practical possibilities of using light scattering methods in industrial conditions (in-process control on the production line)).

## Figures and Tables

**Table 1:** Dimensional characteristic and elemental composition of X39Cr13-base planar cutting blades used in experimental studies.

No.	Sample	Geometry ^1^			Hardness ^2^		Composition ^3^		
	Designation	Length, mm	Width, mm	Thickness, mm	Vickers	Rockwell	Element	Weight, %	Deviation
					**HV ^2^**	**HRC**			
1.	B1-1	459.79	12.33	0.60	737.00	>61.70	Fe	83.00–87.10	±0.02
2.	B1-2	459.49	12.33	0.60	743.00	>61.80	C	0.36–0.42	±0.02
3.	B1-3	459.46	12.30	0.60	741.00	>61.20	Si	1.00	+0.05
4.	B1-4	459.50	12.31	0.60	750.00	>62.50	Mn	1.00	+0.03
5.	B1-5	459.80	12.30	0.60	730.00	>61.40	P	0.04	+0.005
6.	B1-6	459.65	12.31	0.60	737.00	>61.70	S	0.015	+0.003
7.	B1-7	459.15	12.32	0.60	737.00	>61.70	Cr	12.50–14.50	±0.15

Measurement carried out using ^1^ coordinate measuring machine (CMM) Video-Check^®^-IP 250 (Werth Messtechnik GmbH, Löberschütz, Germany) and ^2^ Vickers hardness tester HPO 10 (WPM, Leipzig, Germany); ^3^ according to the EN 10088-1: 2014 standard [28]. Note: HV—Vickers hardness scale, HRC—Rockwell hardness scale.

**Table 2:** The physical and processing properties of X39Cr13 high-carbon martensitic stainless steel.

Country	EU		USA		Germany	France	England		Italy		China		Poland
Standard	EN		-		DIN, WNr	AFNOR	BS		UNI		GB		PN
Designation	X39Cr13		420		X39Cr13	Z40C13	X39Cr13		X40Cr14		4C13		4H13
Physical													
Thermal Expansion	Modulus of Elasticity		Poisson Number		Electrical Resistivity	Electrical Conductivity		Specific Heat		Density		Thermal Conductivity	
10^−6^ K^−1^	GPa		*v*		Ω∙mm^2^/m	S∙m/mm^2^		J/(kg∙K)		kg/dm^3^		W/(m∙k)	
10.5	215		0.27–0.30		0.55	1.82		460		7.7		30	
Processing													
Automated Machining		Machinable		Magnetic		Hammerand Die Forging			ColdForming		Electrical Conductivity		
Yes		Yes		No		Yes			No		No		

**Table 3:** Machining conditions.

Grinding Process		Traverse Surface Grinding		
Grinding Machine		5-axis CNC grinding machine		
Grinding Wheel		5A1 28 × 20 × 10/15 × 14 B126 V180 SV (Inter-Diament, Grodzisk Mazowiecki, Poland)		
Grinding Wheel Dressing Parameters		Dresser: single-grain diamond dresser type M 1010 with a mass of crystal *Q_d_* = 0.75 ct (Inter-Diament, Grodzisk Mazowiecki, Poland), grinding wheel rotational speed while dressing: *n_sd_* = 10,000 min^−1^, dressing allowance: *a_d_* = 0.0125 mm, table feed speed while dressing: *v_fd_* = 10 mm/s, number of dressing passes: *i_d_* = 6		
Grinding Parameters		Grinding wheel peripheral speed: vs. = 55.70 m/s, grinding wheel rotational speed: *n_s_* = 38,000 min^−1^, working engagement (machining allowance): *a_e_* = 0.05 mm, workpiece peripheral speed: *v_w_* = 300–700 mm/min, total grinding time: *t_g tot_* = 210 s		
Workpieces		Working surfaces of planar cutting blades		
GF Delivery Method		Water aerosol delivered by a single spray nozzle		
GF Working Pressure		Supply air pressure: *p* = 0.80 MPa		
GF Type		5% water solution of Climtech M26 synthetic-based water-soluble lubricant (Cimcool Industrial Products Inc., Cincinnati, OH, USA)		
GF Flow Rate		*Q_GF_* = 1050 mL/h		
No.	Sample Designation	Workpiece Peripheral Speed, mm/min	Rotational Speed, min^−1^	Grinding
1	B1-1	300	38,000	Peripheral Grinding
2	B1-2	350		
3	B1-3	400		
4	B1-4	400		Front Grinding
5	B1-5	450		
6	B1-6	500		
7	B1-7	700		

**Table 4:** The observation–measurement instruments used in the experimental studies.

No.	System	Designation	Producer	Components and Features
1.	Optical Stereo Zoom Microscope	SSM- EC2	Schut Geometrische Meettechniek B.V. (Groningen, The Netherlands)	Components (instrument): stand: with round (flattened) column, microscope head: 360°, viewing angle: 45°, eyepieces: 10×, illumination system: incident light/backlight, camera: Micro Cam 10 MP (Bresser GmbH, Rhede, Germany) Features (instrument): magnification: 10–40×, WD = 85 mm Features (camera): type: CMOS, active area size: 6.1 × 4.6 mm, image resolution: 3664 × 2748 pixels
2.	Scanning Electron Microscope	JSM- 5500 LV	JEOL Ltd. (Tokyo, Japan)	Components: detectors: SEI, BEI, specimen stage: eucentric goniometer Features: magnification range: 18–300,000×, vacuum pressure: 10–270 Pa, accelerating voltage: 0.5–30 kV, resolution: for SEI (using HVM) 4.0 nm, for BEI (using LVM) 5.0 nm at pressure of 10 to 270 Pa, evacuation time: for HVM approximately 100 s, for LVM approximately 90 s
				Software: JEOL software v. 1.1 (JEOL Ltd.,Tokyo, Japan)
3.	Dispersive Spectrometer Module	INCAPenta FET-x3	Oxford Instruments (Abingdon, Great Britain)	Components: Si(Li) detector with 30 mm^2^ detecting crystal, number of counts: 4 kcps, resolutions: 129 eV at MnK, 65 eV at FK, 56 eV at CK (line specification according to ISO 15632:2002). The detector was attached to JEOL JSM-5500 LV
				Software: Oxford Instruments software v. 1.4 (Oxford Instruments, Abingdon, UK)
4.	Multisensory Optical Profilometer	Talysurf CLI2000	Taylor-Hobson (Leicester, Great Britain)	Components: laser sensor LK-031 (Keyence Corp., Osaka, Japan) Features (sensor): scanning frequency: 2000 Hz, measuring range: 10 mm, resolution: 1 μm (vertical), 30 μm (lateral), speed: 30 mm/s Features (instrument): measuring capacity: 200× 200 × 200 mm, axis traverse length: 200 mm, axis resolution: 0.5 μm, dimensions: 800 × 800 × 800 mm, measuring speed: 0.5, 1, 5, 10, 15, and 30 mm/s, positioning speed: 30 mm/s
				Software: Talyscan CLI 2000 2.6.1 and TalyMap Silver 4.1.2 (Digital Surf, Besançon, France)

## Nomenclature

ARS	angle-resolved scattering
BEI	backscattered electron imaging
cBN	cubic boron nitride
CMM	coordinate measuring machine
CMOS	complementary metal–oxide–semiconductor
CNC	computerized numerical control
CrN	chromium nitride
DLC	diamond-like coating
GF	grinding fluid
HRC	Rockwell hardness scale
HV	Vickers hardness scale
HVN	high-vacuum mode
LVM	low-vacuum mode
OM	optical microscopy
PSD	plasma-sharpened diamond
PVD	physical vapor deposition
SEI	secondary electron image
SEM	scanning electron microscope
TiAlN	titanium aluminum nitride
TiC	titanium carbide
TiCN	titanium carbon nitride
TiN	titanium nitride
ZrN	zirconium nitride
*a_d_*	dressing allowance, mm
*a_e_*	working engagement (machining allowance), mm
*i_d_*	number of dressing passes
*l*	size (length), mm
*n*	number of samples
*n_s_*	grinding wheel rotational speed, min^−1^
*n_sd_*	grinding wheel rotational speed while dressing, min^−1^
*t_exp_*	exposure time, s
*t_g tot_*	total grinding time, s
*v_fd_*	table feed speed while dressing, mm/s
*v_s_*	grinding wheel peripheral speed, m/s
*v_w_*	workpiece peripheral speed, mm/min
*w*	size (width), mm
*An*	area, mm^2^
*F_max_*	Feret diameter (max. value), mm
*F_min_*	Feret diameter (min. value), mm
*P*	perimeter, mm
*Q_d_*	mass of crystal, ct
*Ra*	arithmetical mean deviation of the roughness profile, μm
*Rq*	root-mean-square deviation of the roughness profile, μm
*Rt*	total height of the profile within a sampling length, μm
*Rz*	maximum height of the profile within a sampling length, μm
*Sa*	arithmetic mean deviation of the surface, μm
*Sq*	root-mean-square deviation of the surface, μm
*St*	total height of the surface, μm
*Sz*	maximum height of the surface, μm
*Ua*	accelerating voltage, kv

## Appendix A

**Table A1:** Basic systematic information of the European plaice (*Pleuronectes platessa*).

Taxonomy						
Kingdom	Phylum	Class	Order	Family	Genus	Species
Animalia	Chordata	Actinopterygii	Pleuronectiforms ^1^	Pleuronectidae	*Pleuronectes*	*P. platessa*
Etymology						
*Pleuronectes → Pleura (gr.)* = side, ribe + *nekton (gr.)* = swimmer						
Binomial name						
*Pleuronectes platessa* Linnaeus, 1758						
Common Name				Synonyms		
Danish Dutch English Estonian Finnish French German Icelandic Latvian	Rødspætte Schol Plaice Merilest Punakampela Plie Scholle Skarkoli Jüras zeltplekste	Norwegian Polish Portuguese Russian Spanish Swedish Alpha code Taxonomic code	Rødspette Gładzica Solha Mopcкaя кaмбaлa Solla Rödspätta PLE 1830200405	*Solea platessa* Rafinesque, 1810:14. *Platessa platessa* Cuvier, 1817:220. *Platessa vulgaris* Cloquet, 1826:403. *Platessa borealis* Faber, 1828:244. *Platessa lata* Cuvier, 1829:339. *Pleuronectes latus*, Valenciennes, 1836-49:300. *Pleuronectes platessa var. baltica*, Nilsson, 1855:616. *Pleuronectes (Platessa) platessa* Danois, 1913:101		

^1^ The flat fishes belong to the order Pleuronectiformes—ray-finned (*Actinopterygii*) demersal fishes. Pleuronectiformes include over 700 species in 11 families (largest are Cynoglossidae, Paralichthyidae, Pleuronectidae, and Soleidae). The Pleuronectidae family contains a group of salt-water species, such as American plaice (*Hippoglossoides platessoides*), European plaice (*Pleuronectes platessa*), scale-eye plaice (*Acanthopsetta nadeshnyi*), and Alaskan plaice (*Pleuronectes quadrituberculatus*).

**Table A2:** General morphological characteristics and basic information (environment, distribution, countries of occurrence) of the European Plaice (*Pleuronectes platessa*).

Description					Environment			
Length, mm			Weight, kg	Age, years	Marine, Brackish, Demersal	Depth, m		Temperature, °C
Max.	Common	Maturity	Max.	Max.		Range	Usually	Usually
1000	500–600	308	7	50		0–200	10–50	2–15
Distribution								
Western Mediterranean, European coasts, White and Barents Seas								
Countries of occurrence								
Europe: Belgium, Denmark, Faroe Islands, France, Germany, Great Britain, Iceland, Ireland, Italy, Latvia, Lithuania, Netherlands, Norway, Portugal, Poland, Russia, Spain, Sweden, Africa: Morocco								
